# Structural and functional ramifications of antigenic drift in recent SARS-CoV-2 variants

**DOI:** 10.1126/science.abh1139

**Published:** 2021-05-20

**Authors:** Meng Yuan, Deli Huang, Chang-Chun D. Lee, Nicholas C. Wu, Abigail M. Jackson, Xueyong Zhu, Hejun Liu, Linghang Peng, Marit J. van Gils, Rogier W. Sanders, Dennis R. Burton, S. Momsen Reincke, Harald Prüss, Jakob Kreye, David Nemazee, Andrew B. Ward, Ian A. Wilson

**Affiliations:** 1 Department of Integrative Structural and Computational Biology, The Scripps Research Institute, La Jolla, CA 92037, USA.; 2 Department of Immunology and Microbiology, The Scripps Research Institute, La Jolla, CA 92037, USA.; 3 Department of Biochemistry, University of Illinois at Urbana-Champaign, Urbana, IL 61801, USA.; 4 Carl R. Woese Institute for Genomic Biology, University of Illinois at Urbana-Champaign, Urbana, IL 61801, USA.; 5 Department of Medical Microbiology and Infection Prevention, Amsterdam University Medical Centers, Location AMC, University of Amsterdam, Amsterdam, Netherlands.; 6 Department of Microbiology and Immunology, Weill Medical College of Cornell University, New York, NY 10021, USA.; 7 Ragon Institute of MGH, Harvard, and MIT, Cambridge, MA 02139, USA.; 8 German Center for Neurodegenerative Diseases (DZNE) Berlin, Berlin, Germany.; 9 Department of Neurology and Experimental Neurology, Charité-Universitätsmedizin Berlin, corporate member of Freie Universität Berlin, Humboldt-Universität Berlin, and Berlin Institute of Health, Berlin, Germany.; 10 Skaggs Institute for Chemical Biology, The Scripps Research Institute, La Jolla, CA 92037, USA.

## Abstract

Neutralizing antibodies (nAbs) elicited against the receptor-binding site (RBS) of the spike protein of wild-type SARS-CoV-2 are generally less effective against recent variants of concern. RBS residues E484, K417 and N501 are mutated in variants first described in South Africa (B.1.351) and Brazil (P.1). We analyzed their effects on ACE2 binding and K417N and E484K mutations on nAbs isolated from COVID-19 patients. Binding and neutralization of the two most frequently elicited antibody families (IGHV3-53/3-66 and IGHV1-2), which can both bind the RBS in alternate binding modes, are abrogated by K417N, E484K, or both. These effects can be structurally explained by their extensive interactions with RBS nAbs. However, nAbs to the more conserved, cross-neutralizing CR3022 and S309 sites were largely unaffected. The results have implications for next-generation vaccines and antibody therapies.

The COVID-19 pandemic has already lasted for over a year, but new infections are still escalating throughout the world. While several different COVID-19 vaccines have been deployed globally, a major concern is the emergence of antigenically distinct SARS-CoV-2 variants of concern (VOCs). In particular, the B.1.1.7 lineage that arose in the UK (*1*) and quickly became dominant, B.1.351 (also known as 501Y.V2) lineage in South Africa (*2*), B.1.1.28 lineage (and its descendant B.1.1.28.1, aka P.1/501Y.V3) in Brazil (*3*), and B.1.232/B.1.427/B.1.429 (aka CAL.20C and CAL.20A) in the United States (*4*) have raised serious questions about the nature, extent and consequences of antigenic drift in SARS-CoV-2. In the receptor-binding site (RBS) of the spike (S) protein receptor-binding domain (RBD), the B.1.1.7 lineage has acquired an N501Y mutation, B.1.351 and P.1 lineages share this mutation along with K417N/T and E484K, whereas the California variants have an L452R mutation that is also present in the Indian variant B.1.617 with E484Q (*5*). E484K has also been detected in a few B.1.1.7 genomes (*1*) (Fig. 1A). We therefore investigated the structural and functional consequences of such mutations on neutralizing antibodies (nAbs) isolated from COVID-19 convalescent patients, and their effect on angiotensin-converting enzyme 2 (ACE2) receptor binding.

**Fig. 1:**
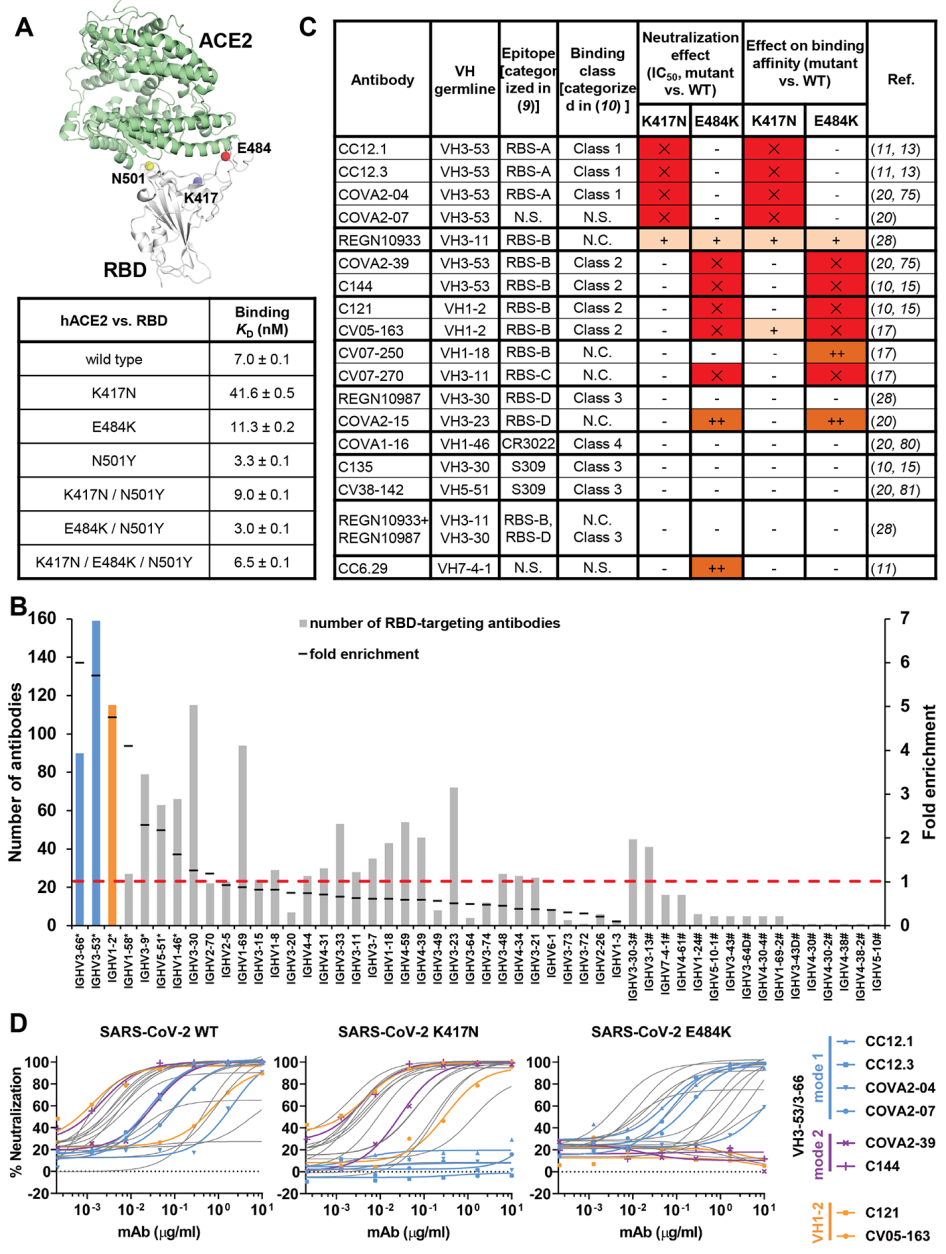
Emergent SARS-CoV-2 variants escape two major classes of neutralizing antibodies. (**A**) Emergent mutations (spheres) in the RBS of B.1.351 and P.1 lineages are mapped onto a structure of SARS-CoV-2 RBD (white) in complex with ACE2 (green) (PDB ID: 6M0J) (*90*). Binding affinities of Fc-tagged human ACE2 against SARS-CoV-2 RBD wild type and mutants were assayed by biolayer interferometry (BLI) experiments. Detailed sensorgrams are shown in fig. S1. (**B**) Distribution of IGHV gene usage. Numbers of RBD-targeting antibodies encoded by each IGHV gene are shown as solid bars. The frequently used IGHV3-53 and IGHV3-66 genes are highlighted in blue, and IGHV1-2 in orange. The IGHV gene usage in 1,593 SARS-CoV-2 RBD-targeting antibodies (*11**, **14*–*44*) compared to healthy individuals (baseline) (*76*) (fold-enrichment) is shown as black lines. ^#^: IGHV gene frequencies in healthy individuals that were not reported in (*76*) are shown with hashtags (^#^). *: IGHV genes that are significantly enriched over the baseline repertoire (*76*) (p < 0.05, one-sample proportion test with Bonferroni correction) are shown with an asterisk (*). A fold-enrichment of one (red dashed line) represents no difference over baseline. (**C**) Effects of single mutations on the neutralization activity and binding affinity of each neutralizing antibody. IC_50_ or *K*_D_ increase that are less than 10-fold are represented by “–”, between 10- and 100-fold as “+”, and greater than 100-fold as “++”. Results in red with “✕” indicate no neutralization activity or binding was detected at the highest amount of IgG used. N.C.: not categorized in the original studies. N.S.: No structure available. (**D**) Neutralization of pseudotyped SARS-CoV-2 virus and variants carrying K417N or E484K mutations. A panel of 17 neutralizing antibodies were tested, including four mode-1 IGHV3-53 antibodies (blue), two mode-2 IGHV3-53 antibodies (purple), and two IGHV1-2 antibodies (orange). The discrepancy between CV05-163 neutralizing SARS-CoV-2 pseudotyped virus (IC_50_ = 0.47 μg/ml) and authentic virus (IC_50_ = 0.02 μg/ml) reported in our previous study (*17*) is possibly due to different systems (pseudovirus vs. authentic virus) and host cells (Hela cells vs. Vero E6 cells) used in these experiments.

N501Y was previously reported to enhance binding to human receptor ACE2 (*6*, *7*). Here, we quantified binding of K417N, E484K, N501Y, and double and triple combinations in the RBD to ACE2 by biolayer interferometry (Fig. 1A and fig. S1). N501Y indeed increased RBD binding to ACE2 compared to wild-type RBD (K_D_ 3.3 nM vs 7.0 nM), whereas K417N substantially reduced ACE2 binding (41.6 nM). E484K slightly reduced binding (11.3 nM). Importantly, N501Y could rescue binding of K417N (9.0 nM), and the triple mutant K417N/E484K/N501Y (as in B.1.351) had similar binding (6.5 nM) to wild type (Fig. 1A and fig. S1). Consistently, K417N/T mutations are associated with N501Y in naturally circulating SARS-CoV-2. Among 585,054 SARS-CoV-2 genome sequences in the GISAID database (March 5, 2021) (*8*), about 95% of K417N/T mutations occur with N501Y, despite N501Y being present in only 21% of all analyzed sequences. In contrast, only 36% of E484K mutations occur with N501Y.

We and others have shown that most SARS-CoV-2 nAbs that target the RBD and their epitopes can be classified into different sites and subsites (*9*–*12*). Certain IGHV genes are highly enriched in the antibody response to SARS-CoV-2 infection, with IGHV3-53 (*11*, *13*–*16*) and IGHV3-66, which differ by only one conservative substitution (V12I), and IGHV1-2 (*11*, *17*, *18*) being the most enriched IGHV genes used among 1,593 RBD-targeting antibodies from 32 studies (*11**, **14*–*44*) (Fig. 1B). We investigated the effects of the prevalent SARS-CoV-2 mutations on neutralization by these multi-donor class antibodies, and the consequences for current vaccines and therapeutics.

K417N and E484K in VOCs B.1.351 and P.1 have been reported to decrease the neutralizing activity of sera as well as neutralizing monoclonal antibodies isolated from COVID-19 convalescent plasma and vaccinated individuals (*45*–*62*). B.1.351 is ~8-14 fold and P.1 is ~2.6-5 fold more resistant to neutralization by convalescent plasma and mRNA vaccinee sera (*63*–*68*). Some variants are able to escape neutralization by some nAbs (e.g., LY-CoV555, 910-30, COVOX-384, S2H58, C671, etc.) while others retain activity (e.g., 1-57, 2-7, mAb-222, S309, S2E12, COV2-2196, C669, etc.) (*50*, *57*, *63*, *66*, *69*, *70*). Here, we selected a representative panel of 17 human nAbs isolated from COVID-19 patients or humanized mice to study the escape mechanism. These nAbs cover all known neutralizing sites on the RBD and include those encoded by V genes that are the most frequently used and also significantly enriched (Fig. 1B). We tested the activity of a panel of nAbs against wild-type (Wuhan strain) SARS-CoV-2 pseudovirus and single mutants K417N and E484K (Fig. 1C). Binding and neutralization of four and five antibodies out of the 17 tested were abolished by K417N and E484K, respectively. Strikingly, binding and neutralization by all six highly potent IGHV3-53 antibodies (*71*) that we tested were abrogated by either K417N (RBS-A/class 1) or E484K (RBS-B/class 2) (Fig. 1, C and D, and fig. S2). In addition, binding and neutralization of IGHV1-2 antibodies was severely reduced for the E484K mutation (Fig. 1, C and D, and fig. S2).

We next examined 54 SARS-CoV-2 RBD-targeting human antibodies with available structures. The antibody epitopes on the RBD can be classified into six sites: four RBS subsites RBS-A, B, C, and D; CR3022 site; and S309 site (fig. S3) (*72*), that are related to the four classes assigned in (*10*) (Fig. 1C). Twenty one of 23 IGHV3-53/3-66 antibodies target RBS-A (Fig. 2A). All IGHV1-2 antibodies with structures to date bind to the RBS-B epitope. A large fraction of antibodies in these two main families make contact with K417, E484 or N501 in their epitopes (Fig. 2A) (*73*). Almost all RBS-A antibodies interact extensively with K417 and N501, whereas most RBS-B and RBS-C antibodies contact E484, and most RBS-C antibodies interact with L452. We also examined the buried surface area (BSA) of K417, E484, and N501 upon interaction with these RBD-targeting antibodies (fig. S3C). The extensive BSA confirmed why mutations at 417 and 484 affect binding and neutralization. Antibodies targeting RBS-D, or the cross-neutralizing S309 and CR3022 sites, are minimally or not involved in interactions with these four RBD mutations (Fig. 2A and fig. S3C).

**Fig. 2:**
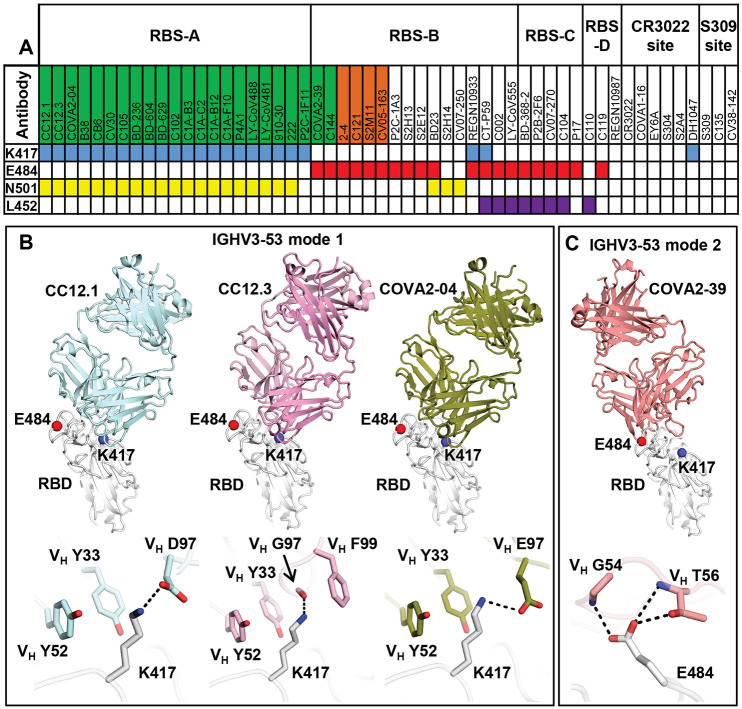
Antibody binding and structures to the wild-type SARS-CoV-2 RBS. (**A**) Antibodies making contact with RBD residues K417, E484 and N501 are represented by blue, red and yellow boxes, respectively (cutoff distance = 4 Å). Antibodies encoded by the most frequently elicited IGHV3-53/3-66 and IGHV1-2 in convalescent patients are shown in green and orange boxes, respectively. Antibodies are ordered by epitopes originally classified in (*9*) with an additional epitope RBS-D that maps to a region in the RBS above or slightly overlapping with the S309 site. Details of the epitope classifications are shown in fig. S3A. Structures of RBD-targeting antibodies that were isolated from patients are analyzed (*91*). (**B** and **C**) Residues that are mutated in recently circulating variants are integral to the binding sites of IGHV3-53 antibodies. Representative structures are shown for (B) IGHV3-53 binding mode 1 [CC12.1 (PDB 6XC3), CC12.3 (PDB 6XC4) (*13*), and COVA2-04 (PDB 7JMO) (*75*)] and (C) binding mode 2 [COVA2-39 (PDB 7JMP) (*75*)]. The SARS-CoV-2 RBD is in white and Fabs in different colors. Residues K417 and E484 are represented by blue and red spheres, respectively. Hydrogen bonds and salt bridges are represented by black dashed lines.

IGHV3-53/3-66 RBD antibodies can adopt two different binding modes (*9*, *10*), which we refer here to as binding modes 1 and 2 (*74*), with distinct epitopes and approach angles (Fig. 2B and fig. S4). All IGHV3-53/3-66 RBD antibodies to date with binding mode 1 have a short CDR H3 of <15 amino acids and bind RBS-A (*13*, *16*, *32*), while those with binding mode 2 have a longer CDR H3 (≥15 amino acids) and target RBS-B (*9*, *10*, *75*). These dual binding modes enhance recognition of this antibody family for the SARS-CoV-2 RBD, although most IGHV3-53/3-66 RBD antibodies adopt binding mode 1 (Fig. 2 and fig. S4). K417 is a key epitope residue for antibodies with IGHV3-53/3-66 binding mode 1 (Fig. 2B and fig. S4). IGHV3-53 germline residues V_H_ Y33 and Y52 make hydrophobic interactions with the aliphatic moiety of K417, and its ε-amino group interacts with CDR H3 through a salt bridge (D97 or E97), hydrogen bond (H-bond), or cation-π interaction (F99) (Fig. 2B). K417N/T would diminish such interactions and, therefore, affect antibody binding and neutralization, providing a structural explanation for K417N escape in IGHV3-53/3-66 antibodies with binding mode 1 (Fig. 1, C and D, Fig. 2B, and fig. S2). In contrast, IGHV3-53 antibodies with binding mode 2 do not interact with RBD-K417 (fig. S4), but with E484 through H-bonds with CDRH2 (Fig. 2C). Consistently, binding and neutralization of IGHV3-53 antibodies with binding mode 2 (fig. S4) are abolished by E484K, but not K417N (Fig. 1, C and D, and fig. S2). Interestingly, unlike most IGHV3-53 antibodies that are sensitive to K417N/T or E484K, a recently discovered IGHV3-53-encoded mAb-222, which binds RBS retains activity against P.1 and B.1.351. The mAb-222 light chain could largely restore the neutralization potency of other IGHV3-53 antibodies, suggesting that light-chain interactions can compensate for loss of binding of K417N/T by the heavy chain (*63*). However, this antibody may represent only a small portion of IGHV3-55/3-66 antibodies that can neutralize VOCs.

Among the IGHV genes used in RBD antibodies, IGHV1-2 is also highly enriched over the baseline frequency in the antibody repertoire of healthy individuals (*76*), and is second only to IGHV3-53/3-66 (Fig. 1B). We compared three structures of IGHV1-2 antibodies, namely 2-4 (*27*), S2M11 (*30*), and C121 (*10*), that target RBS-B. Despite being encoded by different IGK(L)V genes, 2-4 (IGLV2-8), S2M11 (IGKV3-20), and C121 (IGLV2-23) share a nearly identical binding mode and epitope (Fig. 3A). Structural analysis reveals that the V_H_^26^GYTFTG(Y)Y^33^, ^50^W(I)N/S(P)XSXGTX^58^, ^73^TS(I)S/T^76^ motifs are important for RBD binding (fig. S5, A to D). Although only a small part of the epitope interacts with the light chains of 2-4, S2M11, and C121, V_L_ 32 and 91 (n.b. also residue 30 in some antibodies) play an important role in forming a hydrophobic pocket together with V_H_ residues for binding RBD-F486, which is another key binding residue in such classes of antibodies (*9*) (fig. S5, E to I). Three other IGHV1-2 antibodies, 2-43, 2-15, and H4, also bind in a similar mode (*77*), further highlighting structural convergence of IGHV1-2 antibodies in targeting the same RBD epitope. Importantly, all IGHV1-2 antibodies to date form extensive interactions with E484 (Fig. 3A and fig. S3C). In particular, germline-encoded V_H_ Y33, N52 (somatically mutated to S52 in C121) and S54 are involved in polar interactions with the RBD-E484 side chain that would be altered by substitution with Lys (Fig. 3A) and thereby diminish binding and neutralization of IGHV1-2 antibodies against E484K (Fig. 1, C and D, and fig. S2).

**Fig. 3:**
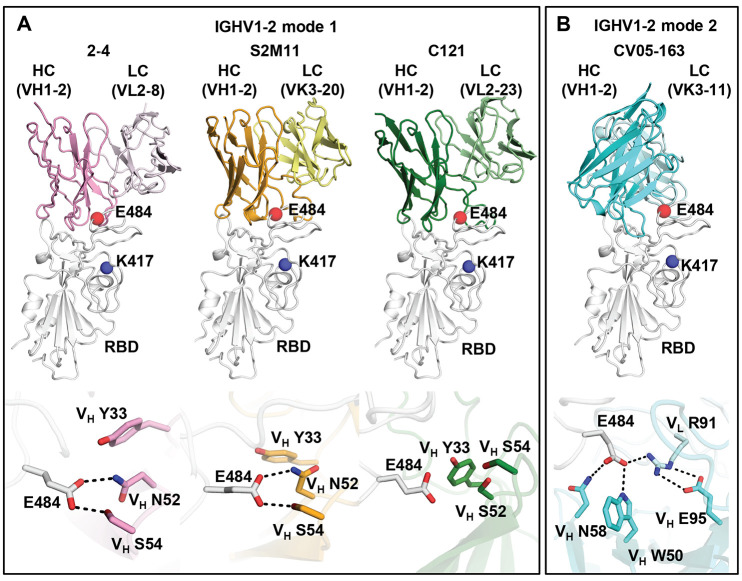
E484 is critical for RBD recognition of IGHV1-2 antibodies. Heavy and light chains of antibody 2-4 (PDB 6XEY) (*27*) are shown in pink and light pink, respectively, S2M11 (PDB 7K43) (*30*) in orange and yellow, and C121 (PDB 7K8X) (*10*) in dark and light green, and CV05-163 in cyan and light cyan. The RBD is shown in white. E484 and K417 are highlighted as red and blue spheres, respectively. Hydrogen bonds are represented by dashed lines. Hydrogen bonds are not shown in the panel of C121 due to the limited resolution (3.9 Å).

We previously isolated another potent IGHV1-2 antibody, CV05-163, targeting the SARS-CoV-2 RBD (fig. S6) from a COVID-19 patient (*17*). CV05-163 likely represents a shared antibody response for IGHV1-2 RBD antibodies across patients (fig. S7). Negative-stain electron microscopy (nsEM) of CV05-163 in complex with the SARS-CoV-2 S trimer illustrates that it can bind in various stoichiometries, including molar ratios of 1:1, 2:1, and 3:1 (Fab to S protein trimer), and can accommodate RBDs in both up- and down-conformations (fig. S8). We also determined a crystal structure of Fab CV05-163 with SARS-CoV-2 RBD and Fab CR3022 to 2.25 Å resolution (Fig. 3B, figs. S9 to S11, and tables S1 and S2) and found that it does indeed bind RBS-B (Fig. 3) and makes extensive interactions with E484 through H-bonds (V_H_ W50 and V_H_ N58) and a salt bridge (V_L_ R91) (Fig. 3B) that explains why CV05-163 binding and neutralization were diminished with E484K (Fig. 1, C and D, and fig. S2). However, CV05-163 is rotated 90° (Fig. 3B) compared to other IGHV1-2 antibodies 2-4, S2M11, and C121 (Fig. 3A). Thus, IGHV1-2 antibodies, akin to IGHV3-53/66 (*75*), can engage the RBD in two different binding modes, both of which are susceptible to escape by E484K, but not by K417N (Fig. 1, C and D).

A further group of antibodies target the back side of the RBS ridge (RBS-C) (*9*). To date, five nAbs isolated from COVID-19 patients are known to bind RBS-C: CV07-270 (*17*), BD-368-2 (*38*), P2B-2F6 (*18*), C104 (*10*), and P17 (*78*). These RBS-C nAbs also interact with E484 (Fig. 4A), mainly through an arginine in CDRH3, suggesting that E484K may adversely impact RBS-C antibodies. Indeed, binding and neutralization by CV07-270 was abrogated by E484K (Fig. 1C and fig. S2). Intriguingly, these five RBS-C antibodies are encoded by five different IGHV genes (*79*), but target a similar epitope with similar angles of approach. In addition, neutralization by REGN10933, a potent antibody used for therapeutic treatment, was reduced to a less extent by K417N and E484K (Fig. 1C) (*28*). REGN10933 binds at a slightly different angle from RBS-A antibodies and other RBS-B antibodies. K417 then interacts with CDRs H1 and H3 of REGN10933, whereas E484 contacts CDRH2 (fig. S12). Overall, our results demonstrate that RBS mutations K417N and E484K can either abolish or extensively reduce the binding and neutralization of several major classes of SARS-CoV-2 RBD antibodies.

**Fig. 4:**
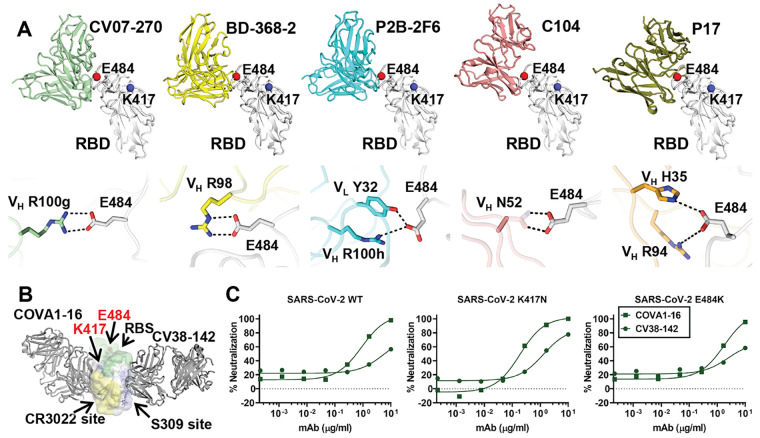
Antibodies targeting other major antigenic sites are differentially affected by mutations in recent variants. (**A**) Interactions between RBS-C antibodies and SARS-CoV-2 RBD. The RBD is shown in white with E484, K417 represented as red and blue spheres, respectively. The various antibodies illustrated are in different colors. Only the variable domains are shown for clarity. Hydrogen bonds and salt bridges to E484 are represented by dashed lines. Published structures with PDB IDs 6XKP (*17*), 7CHF (*38*), 7BWJ (*18*), 7K8U (*10*), and 7CWN (*78*) are used to depict structures of SARS-CoV-2 RBD with CV07-270, BD-368-2, P2B-2F6, C104, and P17, respectively. The electron density for the full side chain of V_H_ N52 was not well resolved in the 3.8-Å structure of C104 in complex with SARS-CoV-2 S. The full side chain is modeled here and shown as transparent sticks to illustrate a possible interaction with E484. (**B**) Cross-neutralizing antibodies to the RBD are not affected by E484 and K417 mutations. COVA1-16 targets the CR3022 cryptic site (yellow) (*80*) and CV38-142 targets the S309 proteoglycan site (blue) (*81*) to the RBD. Glycans at the N343 glycosylation site are represented by sticks. The RBS surface is shown in green. E484 and K417 are highlighted as red and blue spheres, respectively. (**C**) Neutralization of CV38-142 and COVA1-16 against SARS-CoV-2 wild type, K417N or E484K pseudoviruses.

Two other non-RBS sites that are distant from K417 and E484 have been repeatedly shown to be neutralizing sites on the SARS-CoV-2 RBD, namely the CR3022 cryptic site and S309 proteoglycan site (*9*) (Fig. 1C and Fig. 4B). Antibodies from COVID-19 patients can neutralize SARS-CoV-2 by targeting the CR3022 site, including COVA1-16 (*80*), S304, S2A4 (*31*), and DH1047 (*41*). Recently, we isolated antibody CV38-142 that targets the S309 site (*81*). Antibodies targeting these two epitopes are often cross-reactive with other sarbecoviruses, as these sites are more evolutionarily conserved compared to the RBS. To test the effect of the K417N and E484K mutations on nAbs that target the S309 and CR3022 sites, we assessed binding and neutralization by CV38-142 and COVA1-16 to SARS-CoV-2. Both mutations have minimal effect on these antibodies (Fig. 1C and Fig. 4C).

The most potent neutralizing antibodies to SARS-CoV-2 generally tend to target the RBS (table S3), as they directly compete with receptor binding. Such RBS antibodies often interact with K417, E484, or N501, which are located in the RBS, and are therefore sensitive to RBS mutations in the VOCs. On the other hand, antibodies targeting the CR3022 and S309 sites are often less potent, but are less affected by the VOCs, as their epitopes do not contain mutated residues. In fact, recent studies have shown that sera from convalescent or vaccinated individuals can retain neutralization activity, albeit reduced, against the mutated variants (*48*, *50*, *82*), which is possibly due to antibodies targeting other epitopes including the CR3022 and S309 sites. Thus, the CR3022 and S309 sites are promising targets to avoid interference by SARS-CoV-2 mutations observed to date.

As SARS-CoV-2 continues to circulate in humans and increasing numbers of COVID-19 vaccines are administered, herd immunity to SARS-CoV-2 should be approached locally and globally. However, as with other RNA viruses, such as influenza and HIV (*83*), further antigenic drift is anticipated in SARS-CoV-2. Intra-host antigenic drift has also been observed in an immunosuppressed COVID-19 patient who had low titers of neutralizing antibodies that allowed emergence of N501Y and E484K mutations (*84*). While antibody responses elicited by the wild-type lineage that initiated the COVID-19 pandemic have been well characterized in natural infection (*11*, *14*–*18*, *20*, *23*, *26*, *27*, *29*) and vaccination (*50*, *85*–*87*), data on the immune response to VOCs are now only starting to emerge (*88*) and will inform on the similarity and differences in the antibodies elicited. Since SARS-CoV-2 is likely to become endemic (*89*), the findings here and in other recent studies can be used to fast-track development of more broadly effective vaccines and therapeutics.

## Supplementary Materials

science.sciencemag.org/cgi/content/full/science.abh1139/DC1 Materials and Methods Figs. S1 to S12 Tables S1 to S3 References (*92*–*110*) MDAR Reproducibility Checklist
